# A Potent, Broad-Spectrum Antiviral Agent that Targets Viral Membranes

**DOI:** 10.3390/v2051106

**Published:** 2010-05-04

**Authors:** Jason A. Wojcechowskyj, Robert W. Doms

**Affiliations:** Department of Microbiology, University of Pennsylvania, Philadelphia PA 19104, USA; E-Mail: doms@mail.med.upenn.edu

## Abstract

Commentary on Wolf, M.C.; Freiberg, A.N.; Zhang, T.; Akyol-Ataman, Z.; Grock, A.; Hong, P.W.; Li, J.; Watson, N.F.; Fang, A.Q.; Aguilar, H.C.; *et al.* A broad-spectrum antiviral targeting entry of enveloped viruses. *Proc. Natl. Acad. Sci. U. S. A.* **2010**, *107*, 3157–3162.

In contrast to several widely used antibiotics, antiviral drugs typically target a single virus. The rising number of diverse emerging and endemic viral diseases that pose a significant health risk to human populations underscores the need to develop broad-spectrum antivirals. However, the few broad-spectrum antivirals that are licensed have limited potency and are poorly tolerated [1]. Theoretically, a broad-spectrum antiviral compound could target a conserved host pathway or protein that supports infection by multiple viruses or a common viral structural feature. As an example, host proteases are potential targets for broad-spectrum antivirals since many viruses such as Ebola, reovirus and coronavirus all require host proteases for entry [2–4]. In addition, the host enzyme IMP dehydrogenase, the target of ribavirin, is required for the replication of several RNA and DNA viruses [1]. Currently in phase II clinical trials for treatment of influenza, T-705 (favipiravir), also inhibits several RNA viruses - likely through selective inhibition of the viral RNA-dependent RNA polymerase [5]. In a recent PNAS study, Wolf *et al.* describe the discovery and characterization of a promising broad-spectrum antiviral compound, LJ001, which shows activity against an impressive number of enveloped viruses [6].

LJ001 is an aryl methyldiene rhodanine derivative that was found during a high-throughput cell-based screen for inhibitors of Nipah virus (NiV) entry using a vesicular stomatitis virus (VSV) luciferase reporter pseudotype system. LJ001 caused minimal cellular toxicity at *in vitro* inhibitory concentrations. The authors employed several independent approaches to show that LJ001 acts at the level of virus entry. For example, inhibition was only achieved if LJ001 was added before or during virus absorption onto cells - if it was added after virus adsorption, infection occurred normally. A beta-lactamase content mixing assay further demonstrated a role during entry, though a binding assay showed that viral attachment occurred normally, thus implicating virus-membrane fusion as a potential target.

To this point, the activity of LJ001 resembled that of other inhibitors that act at very early stages of the viral lifecycle. However, it became evident that LJ001 showed antiviral activity not just against Nipah virus, but also against an impressive array of viruses including representatives of the filoviridae, orthomyxoviridae, arenaviridae, bunyaviridae, paramyxoviridae, flaviviridae, retroviridae, poxviridae and rhbdoviridae, but not against members of the adenoviridae, picornoviridae and reoviridae. As a result, its antiviral activity was independent of the viral glycoprotein responsible for mediating virus entry. The only unifying, structural feature evident amongst the diverse viruses that were inhibited by LJ001 is the presence of a viral membrane: LJ001 inhibited all enveloped viruses tested, but failed to inhibit nonenveloped viruses. Aided by its intrinsic fluorescence, the authors showed that LJ001 associates with liposomes, and that liposomes can compete with NiV-pseudotyped VSV for LJ001 unless virions were pretreated with LJ001 before addition of liposomes. The authors suggest that LJ001 intercalates into viral membranes in an essentially irreversible manner, preventing membrane fusion in a manner that is independent of the viral membrane fusion protein. To further support a proposed viral glycoprotein-independent mechanism of action, the authors attempted to generate resistant HIV mutants through serial passaging, yet resistant virus failed to develop after four weeks. Longer passaging studies as well as the use of additional strains of the viruses tested thus far may provide clues for a mechanism of action and address the important issue of whether resistance can be generated. Another unanswered question is whether LJ001 intercalates preferentially into viral membranes, or whether it associates equally well with any lipid bilayer regardless of specific protein or lipid content. Without some degree of membrane specificity, it is difficult to envisage how LJ001 will achieve high enough levels *in vivo* to prove effective. If systemic administration becomes impractical, a topical formulation may be feasible, as with the anti-herpesvirus drug Docosanol (Abreva) [7]. Importantly, the association of LJ001 with viral membranes was potent enough to protect mice infected with lethal doses of Ebola or Rift Valley Fever virus, but only when virions were pre-treated with LJ001 prior to inoculation of the animals.

Though LJ001 associates with both viral and host membranes, it is only the viral membrane whose function appears to be impaired: LJ001 inhibits virus-cell fusion, but it fails to inhibit cell-cell fusion reactions mediated by the same viral glycoprotein. The authors hypothesize that this is due to the fact that host membranes are continually remodeled and can repair themselves by metabolizing or extracting membrane-active agents, thus escaping gross membrane perturbation [8]. Consistent with this, synergistic disruption of plasma membrane integrity was observed when cells were exposed to an inhibitor of fatty acid synthesis (TOFA) and LJ001. This again suggests that simply associating with lipid membranes is not sufficient for antiviral activity, though the precise mechanism has not yet been determined. One mechanism by which LJ001 could potentially prevent fusion is by altering membrane curvature, as a variety of non-bilayer lipids that impact membrane curvature have been shown to inhibit fusion [9].

Other membrane targeting broad-spectrum antivirals have been developed with varying degrees of success. Cosalane is thought to associate with viral membranes and showed activity against HIV and some herpes viruses *in vitro*; however, due to its hydrophobic properties, efforts are focused on maximizing bioavailability and tissue clearance [10,11]. Early studies with Docosanol (Abreva), a saturated alcohol, showed activity against several enveloped viruses and was found to bind to viral membranes [12]. It is currently licensed as a topical cream for treatment of herpes cold sores [7]. Arbidol is an indole derivative that is currently licensed in Russia to treat acute respiratory infections. Like LJ001, arbidol has a strong affinity for membranes and shows a wide spectrum of activity against not only several enveloped but also some non-enveloped viruses [13]. While the mechanism of action of arbidol is not entirely clear, a recent study with HCV shows some similarities with LJ001 [14].

The discovery of LJ001 serves as an important proof of principle for a more global and unbiased approach towards the screening of antiviral compounds. While traditional *in vitro* enzymatic screening approaches have led to the development of potent and highly specific antiviral agents, cell-based screens such as undertaken by Wolf *et al.* may prove even more successful in drug discovery in part because biological activity and toxicity are upfront criteria in choosing lead compounds – factors which often halt the progression of otherwise promising compounds – while sidestepping the expensive undertaking of biological target validation that must precede *in vitro*-based screens [15]. Live-cell screening approaches also afford the opportunity to identify antiviral agents that target any aspect of the virus lifecycle, including compounds that target conserved host cell components rather than viral proteins themselves. The unprecedented activity of LJ001 against numerous enveloped viruses raises the possibility that effective, broad-spectrum antivirals can be identified.
